# Supplementary material for: The nuclear receptor HNF4 drives a brush border gene program conserved across murine intestine, kidney, and embryonic yolk sac
Source: Nat Commun. 2021 May 17;12:2886. doi: 10.1038/s41467-021-22761-5 (PMC8129143; doi:10.1038/s41467-021-22761-5)
Supplement: Supplementary file 1 — Supplementary Information [file 41467_2021_22761_MOESM1_ESM.pdf]

Supplementary Information

The nuclear receptor HNF4 drives a brush border gene program conserved across murine intestine, kidney, and embryonic yolk sac

Lei Chen[1,2*], Shirley Luo[1], Abigail Dupre[1], Roshan P. Vasoya[1], Aditya Parthasarathy[1], Rohit Aita[1], Raj Malhotra[1], Joseph Hur[1], Natalie H. Toke[1], Eric Chiles[2], Min Yang[1], Weihuan Cao[1], Juan Flores[3], Christopher E. Ellison[1], Nan Gao[3], Amrik Sahota[1], Xiaoyang Su[2,4], Edward M. Bonder[3], Michael P. Verzi[1,2,5*]

[1]Department of Genetics, Human Genetics Institute of New Jersey, Rutgers University, Piscataway, NJ 08854, USA
[2]Rutgers Cancer Institute of New Jersey, Rutgers University, New Brunswick, NJ 08903, USA
[3]Department of Biological Sciences, Rutgers University, Newark, NJ 07102, USA
[4]Department of Medicine, Rutgers-Robert Wood Johnson Medical School, New Brunswick, NJ, 08901, USA
[5]Rutgers Center for Lipid Research, New Jersey Institute for Food, Nutrition & Health, Rutgers University, New Brunswick, NJ 08901, USA

*Correspondence: lchen@dls.rutgers.edu (L.C.); verzi@biology.rutgers.edu (M.P.V.)

Supplementary Figures 1-12

Supplementary Table 1

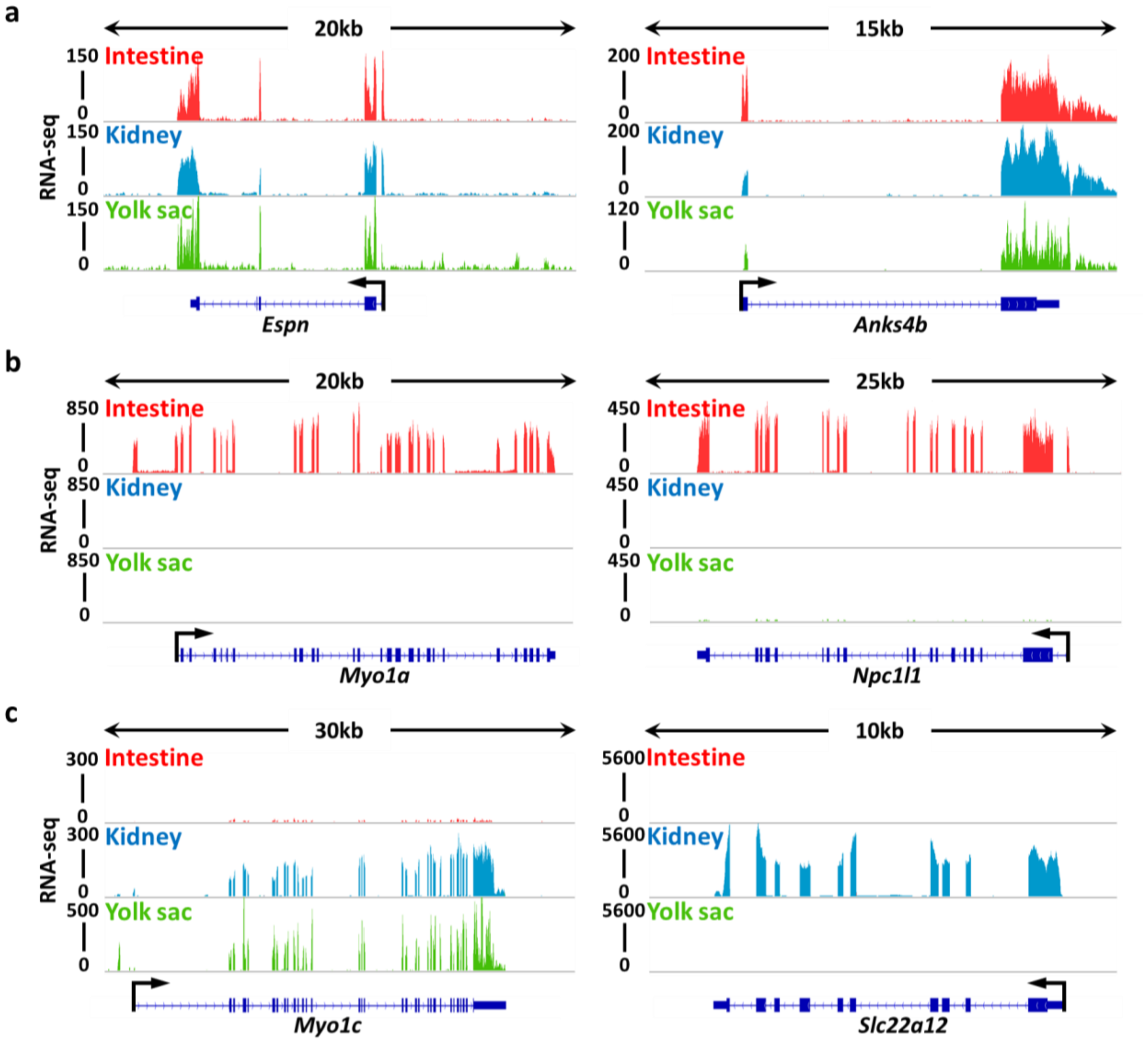

Supplementary Fig 1. Examples of brush border gene expression levels in different tissues. (a) RNA-seq tracks of common brush border genes in intestine, kidney and yolk sac. **(b-c)** RNA-seq tracks of tissue specific brush border genes.

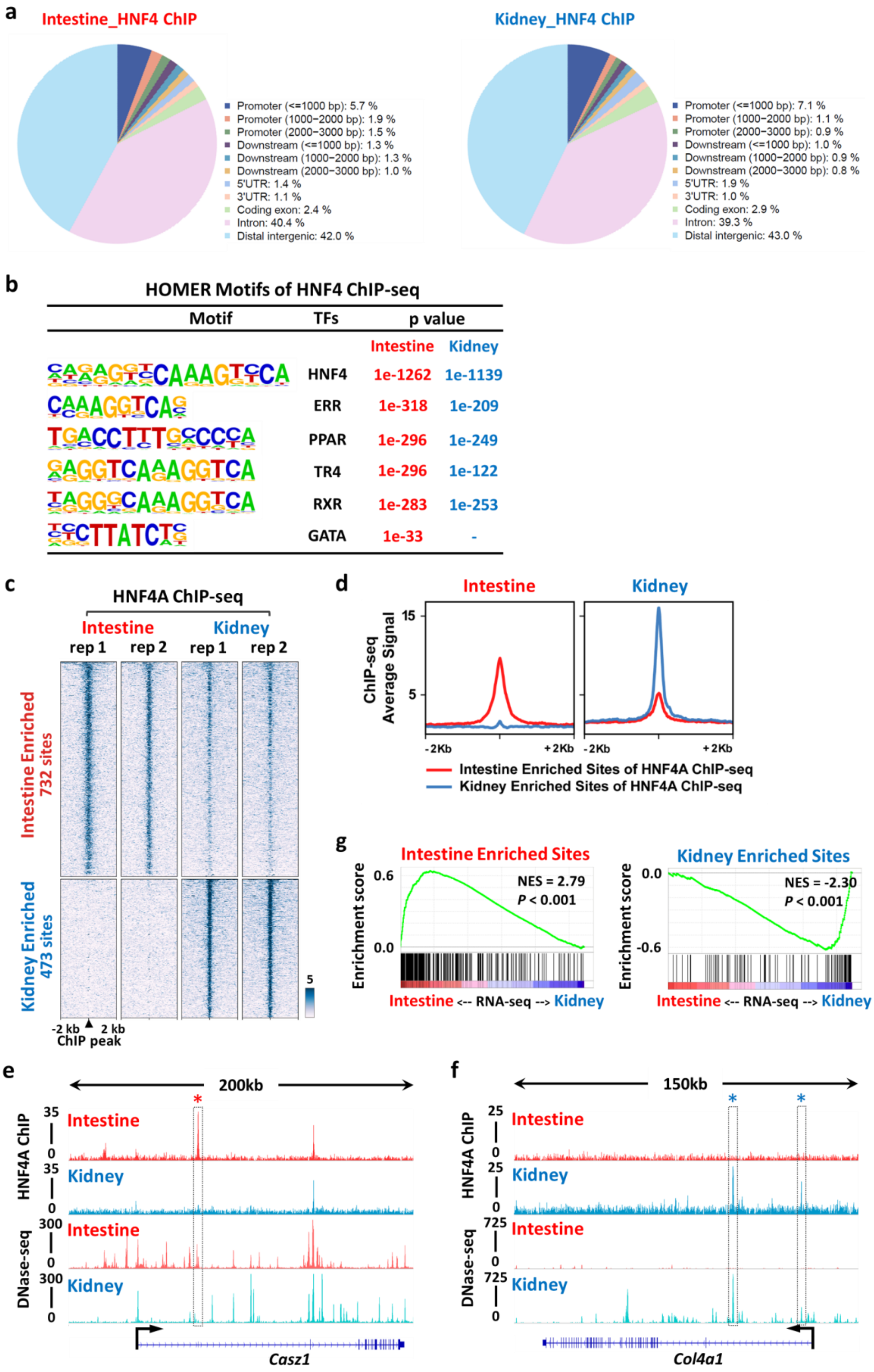
a
Intestine_HNF4 ChIP
Promoter (<=1000 bp): 5.7 %
Promoter (1000−2000 bp): 1.9 %
Promoter (2000−3000 bp): 1.5 %
Downstream (<=1000 bp): 1.3 %
Downstream (1000−2000 bp): 1.3 %
Downstream (2000−3000 bp): 1.0 %
5'UTR: 1.4 %
3'UTR: 1.1 %
Coding exon: 2.4 %
Intron: 40.4 %
Distal intergenic: 42.0 %
Kidney_HNF4 ChIP
Promoter (<=1000 bp): 7.1 %
Promoter (1000−2000 bp): 1.1 %
Promoter (2000−3000 bp): 0.9 %
Downstream (<=1000 bp): 1.0 %
Downstream (1000−2000 bp): 0.9 %
Downstream (2000−3000 bp): 0.8 %
5'UTR: 1.9 %
3'UTR: 1.0 %
Coding exon: 2.9 %
Intron: 39.3 %
Distal intergenic: 43.0 %
b
HOMER Motifs of HNF4 ChIP-seq
Motif
TFs
p value
Intestine
Kidney
HNF4 1e-1262 1e-1139
ERR 1e-318 1e-209
PPAR 1e-296 1e-249
TR4 1e-296 1e-122
RXR 1e-283 1e-253
GATA 1e-33 -
c
HNF4A ChIP-seq
Intestine
Kidney
rep 1
rep 2
rep 1
rep 2
Intestine Enriched
732 sites
Kidney Enriched
473 sites
5
-2 kb
2 kb
ChIP peak
d
Intestine
Kidney
ChIP-seq
Average Signal
15
5
-2Kb
+2Kb
-2Kb
+2Kb
Intestine Enriched Sites of HNF4A ChIP-seq
Kidney Enriched Sites of HNF4A ChIP-seq
g
Intestine Enriched Sites
Enrichment score
0.6
0.0
NES = 2.79
P < 0.001
Intestine <-- RNA-seq --> Kidney
Kidney Enriched Sites
0.0
-0.6
NES = -2.30
P < 0.001
Intestine <-- RNA-seq --> Kidney
e
200kb
HNF4A ChIP
35
0
Intestine
35
0
Kidney
DNase-seq
300
0
Intestine
300
0
Kidney
Casz1
f
150kb
HNF4A ChIP
25
0
Intestine
25
0
Kidney
DNase-seq
725
0
Intestine
725
0
Kidney
Col4a1

Supplementary Fig 2. HNF4 binding analysis in the intestine and kidney. (a) CEAS analysis of HNF4 ChIP-seq binding distribution. HNF4 mainly occupies regions far from transcription start sites. **(b)** HOMER motif analysis of HNF4 binding sites called by MACS (intestinal epithelium MACS $P \leq 10^{-3}$; kidney MACS $P \leq 10^{-5}$). Statistical tests were embedded in the MACS and HOMER packages. **(c)** Heatmaps and **(d)** SitePro plots show ChIP-seq signals of differentially bound sites (DiffBind analysis, FDR < 0.01) between HNF4A ChIP-seq in intestine and kidney (n = 2). **(e-f)** IGV tracks of HNF4A ChIP-seq (GSE112946 and GSE47192) and DNase-seq (GSE57919 and GSE51336) at differentially bound loci between intestine and kidney (examples identified from **Supplementary Fig. 2c**). **(g)** GSEA reveals that genes that are differentially bound by HNF4A in intestine or kidney are highly expressed in the corresponding tissues (Kolmogorov-Smirnov test, one-sided for positive and negative enrichment scores, P < 0.001). Statistical tests were embedded in the GSEA.

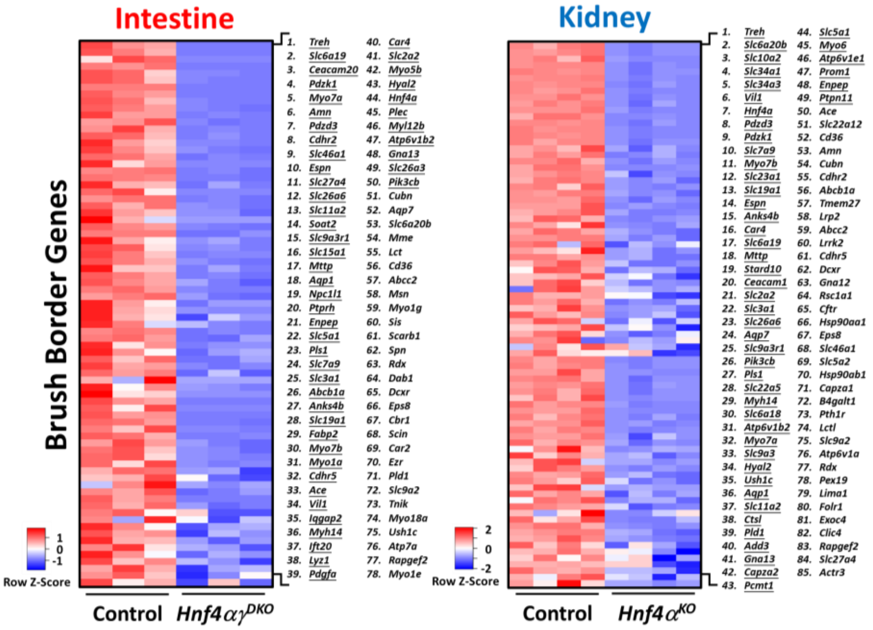

Supplementary Fig 3. Brush border genes are compromised upon HNF4 loss. RNA-seq data of downregulated brush border genes (FDR < 0.05, n = 3 WT & 3 mutant intestine samples; n = 4 WT & 4 mutant kidney samples) are visualized by heatmaps. Genes with underlines are within 30 kb of HNF4 binding sites and identified as HNF4 direct targets (Intestine: MACS $P \leq 10^{-3}$; Kidney: MACS $P \leq 10^{-5}$).

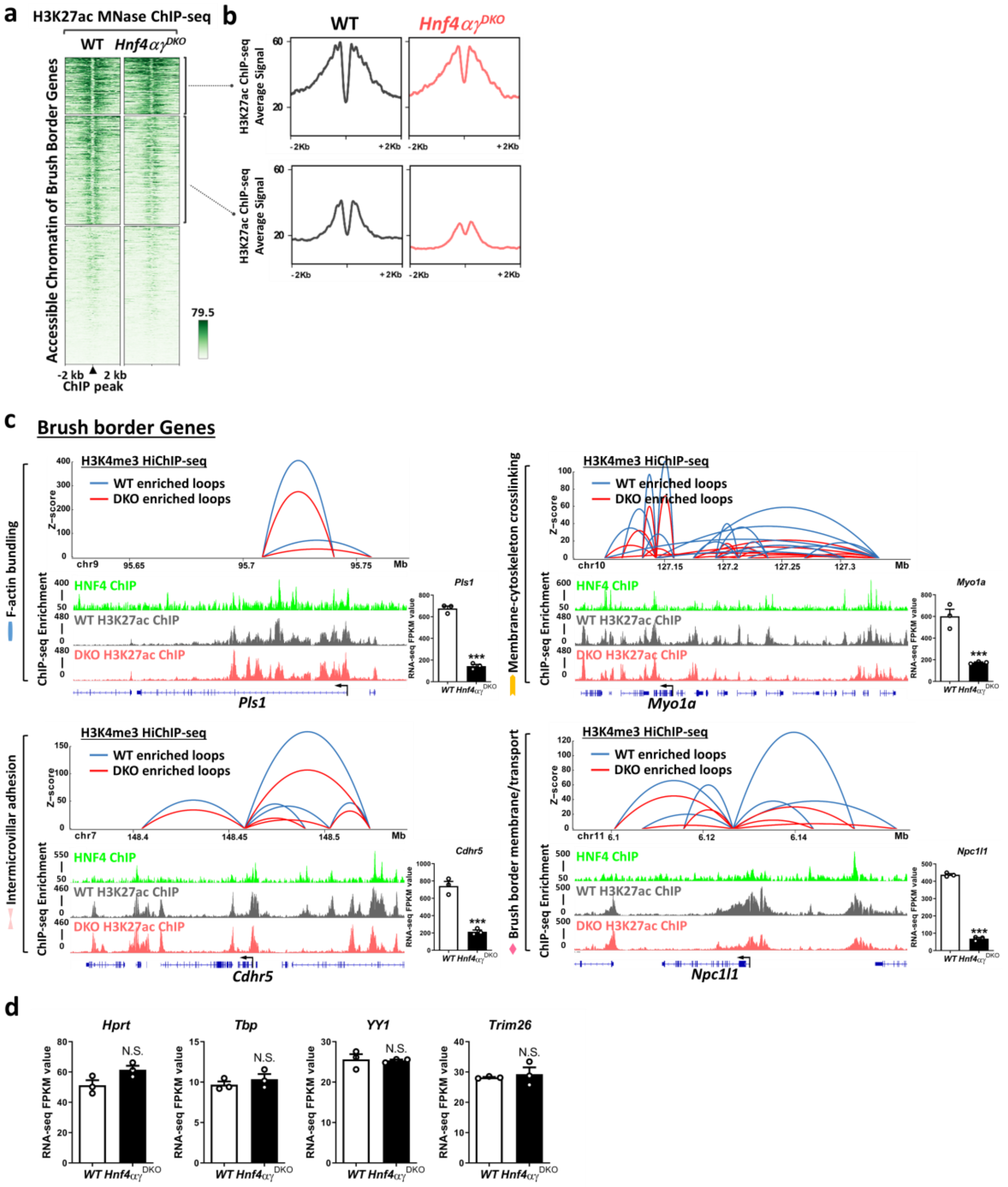

Supplementary Fig 4. Active chromatin markers are compromised at accessible chromatin regions of brush border genes upon HNF4 loss in the intestine. (a) Heatmap and **(b)** average signals of merged H3K27ac ChIP-seq (WT vs *Hnf4*$\alpha\gamma^{DKO}$; n = 2 biological replicates) at accessible chromatin regions of brush border genes (+/- 50 kb of TSSs, enhancers). TSSs: transcription start sites. Decreased chromatin loop formation at loci of **(c)** brush border genes are observed upon HNF4 loss. H3K4me3 HiChIP-seq was done in villus cells of *Hnf4*$\alpha\gamma^{DKO}$ and their littermate controls. Differential loops (DEseq2 $P < 0.05$) are visualized by Sushi package for the loops with q ≤ 0.0001 and counts ≥ 8 (combined 2 replicates). Bar charts show transcript levels of brush border genes. The data are presented as mean ± SEM (RNA-seq: n = 3 biological replicates, Cuffdiff FDR < 0.001***). H3K4me3 HiChIP-seq: n = 2 biological replicates; H3K27ac ChIP-seq: n = 2 biological replicates; HNF4 ChIP-seq: n = 2 biological replicates. **(d)** The transcript levels of housekeeping/non-brush border genes. The data are presented as mean ± SEM (n = 3 biological replicates; N.S.: not significant). Statistical tests were embedded in Cuffdiff.

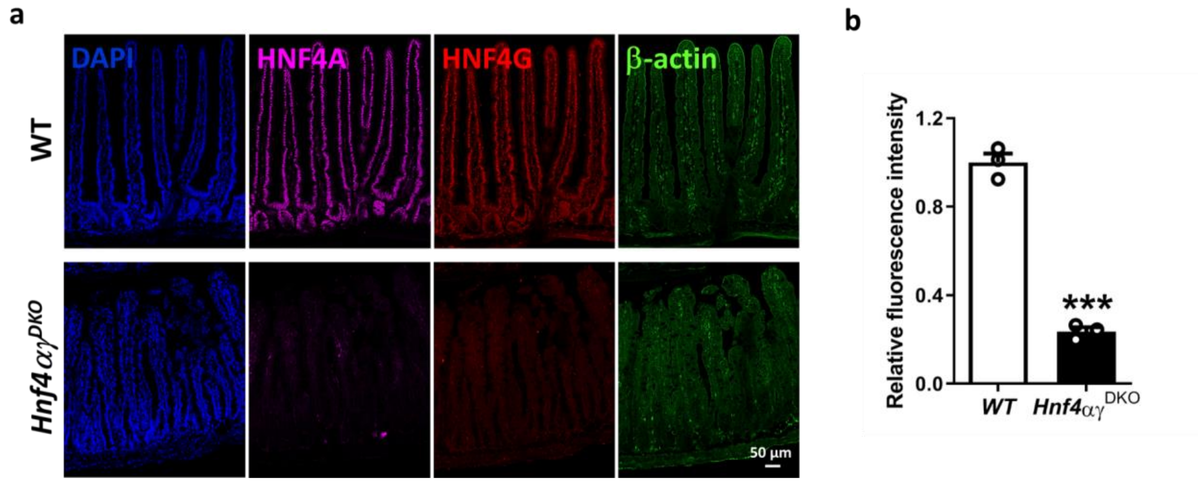

Supplementary Fig 5. Co-staining of HNF4A, HNF4G and β-actin in the intestine. **(a)** Individual channels of immunofluorescence co-staining in **Fig. 3c**. **(b)** Quantification of fluorescence intensity of β-actin (brush border marker). The data are presented as mean ± SEM (n = 3 biologically independent mice; Student's *t* test, two-sided at $P < 0.001$ ***).

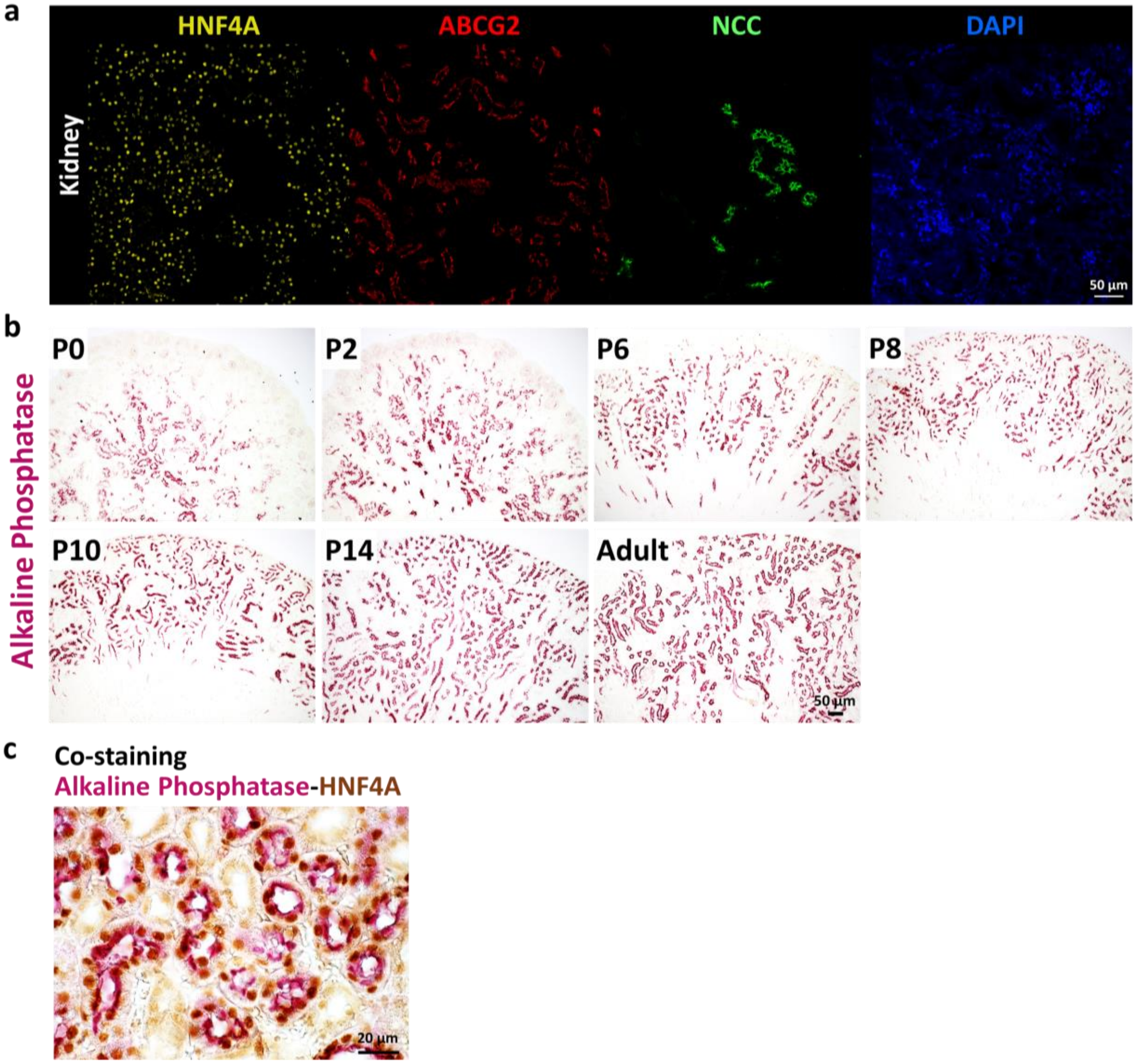

Supplementary Fig 6. Histology of mouse kidney. (a) Individual channels of immunofluorescence co-staining (n = 3 biologically independent mice) in **Fig. 4f**. ABCG2: ATP-binding cassette transporter (proximal tubule marker); NCC: sodium chloride co-transporter (a distal tubule marker). **(b)** Increased alkaline phosphatase activity in mouse kidney was observed over developmental time. Kidney tissues were collected at Day0 (P0), Day2 (P2), Day 6 (P6), Day8 (P8), Day 10 (P10), Day 14 (P14) and 8 weeks (adult) after birth (n = 2 biologically independent mice). **(c)** Co-staining of alkaline phosphatase and HNF4A in adult kidney (n = 3 biologically independent mice).

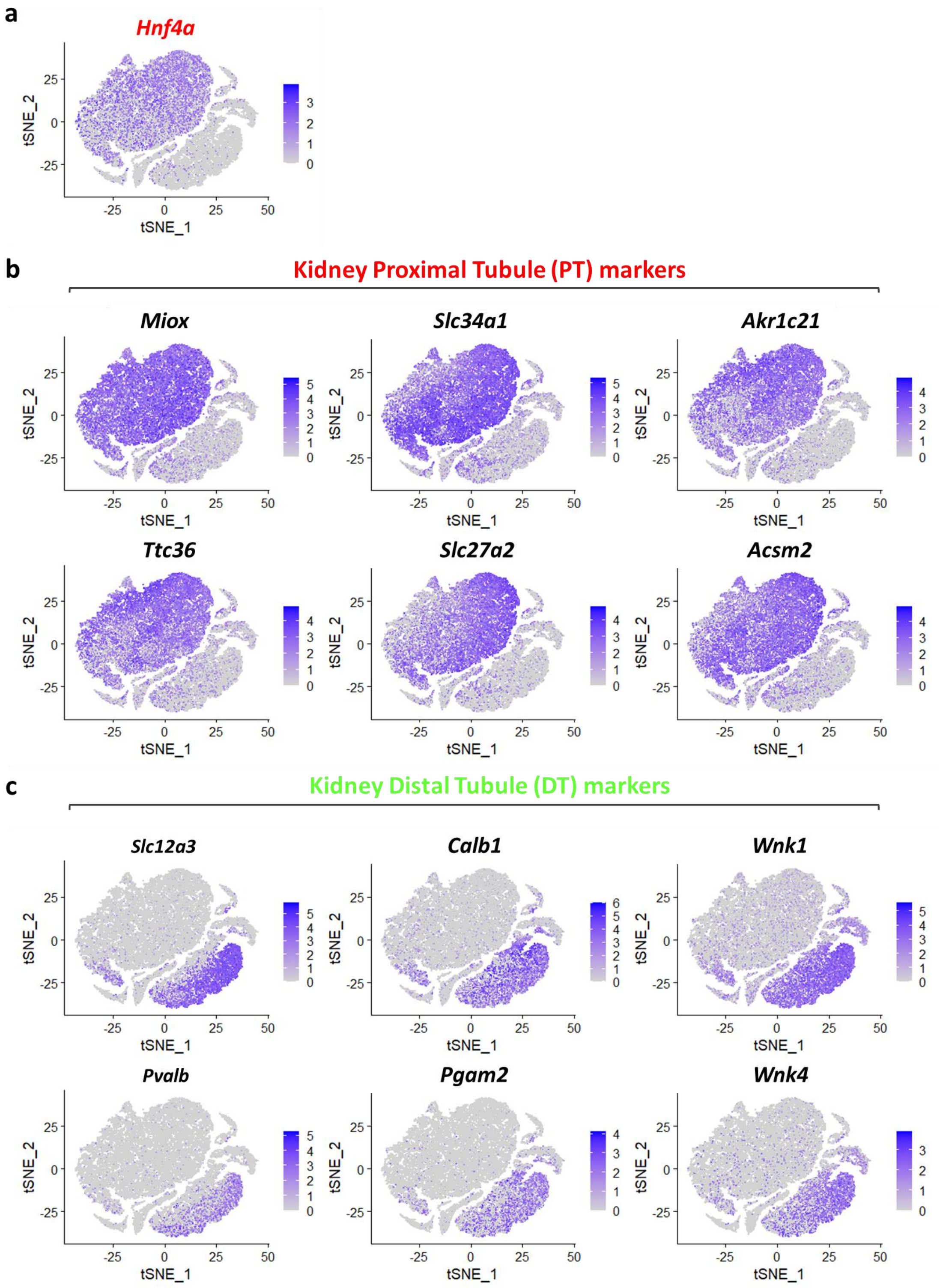

Supplementary Fig 7. HNF4A transcripts are restricted to proximal tubule cells. A single cell RNA-seq study has identified 16 distinct cell types of mouse kidney and their cell type-specific markers, including proximal tubules and distal tubules. These scRNA-seq data (GSE107585) were re-analyzed and visualized using the Seurat package. Cell populations within mouse kidney were defined by markers known to be expressed in kidney proximal tubule cells and distal tubule cells. *Hnf4a* **(a)** is co-expressed with kidney proximal tubule markers **(b)** but not kidney distal tubule markers **(c)**.

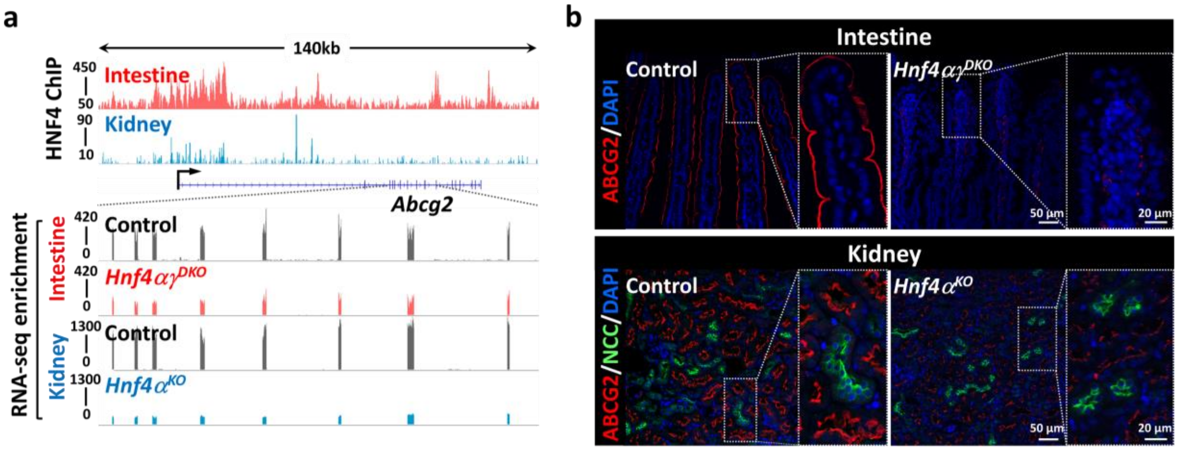

Supplementary Fig 8. Example of HNF4 regulation of a brush border gene. (a) HNF4 ChIP-seq (n = 2 in each tissue) and RNA-seq tracks (Intestine: n = 3; Kidney: n = 4) at *Abcg2* gene locus. **(b)** ABCG2, an ATP-binding cassette transporter, is localized at the apical brush border of intestine and kidney in WT control mice, and compromised upon HNF4 loss (Intestine: 4 days after tamoxifen treatment; Kidney: 7 days after tamoxifen treatment). Unlike the proximal tubules (marked by ABCG2), the distal tubules (marked by NCC) are less affected in *Hnf4*α^{KO} kidney, as evidenced by immunofluorescence confocal microscopy (n = 3 biologically independent mice). The high-magnification images are shown in the boxed insets.

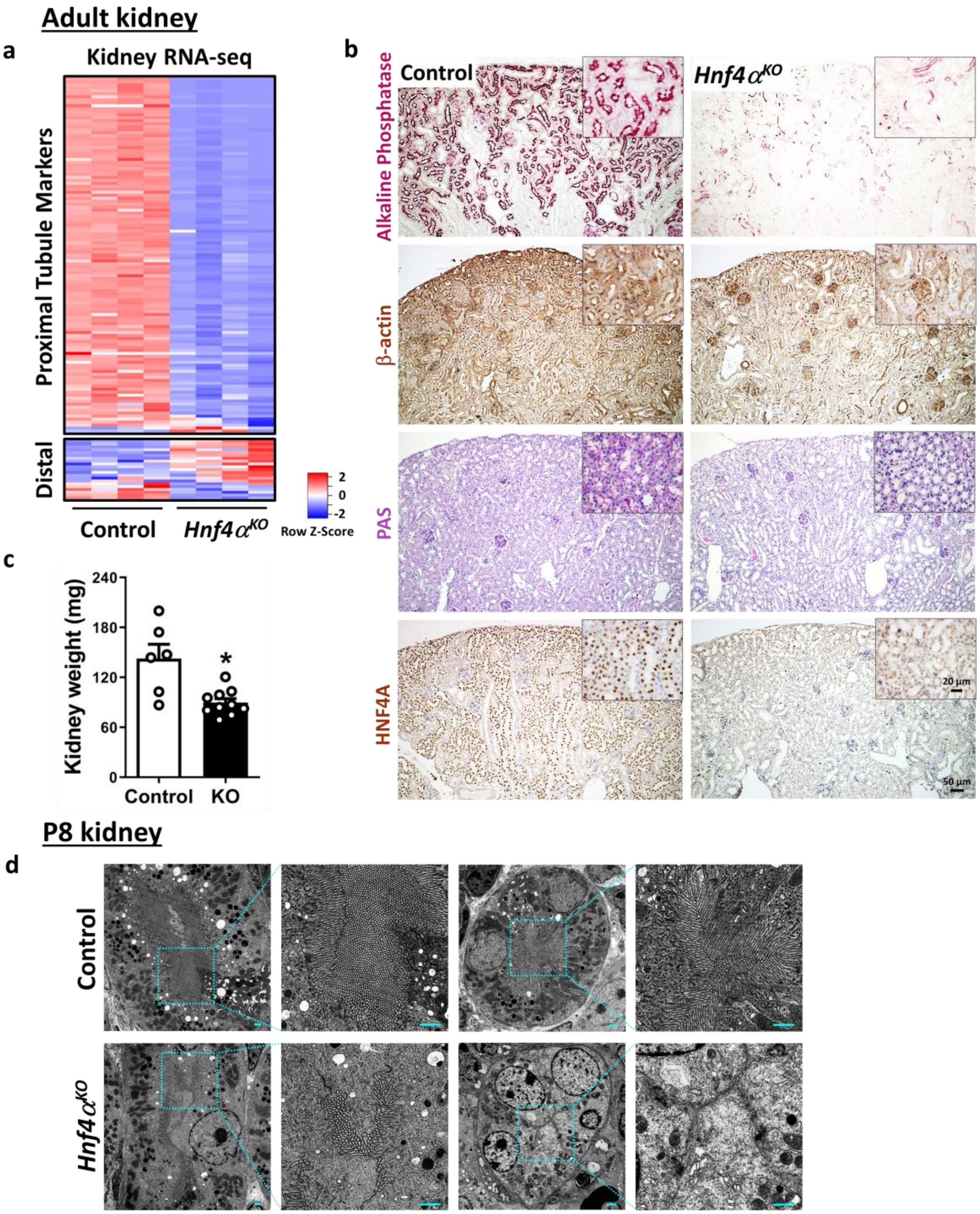

Supplementary Fig 9. HNF4A is required for kidney proximal tubules. (a) Heatmap of kidney RNA-seq data reveals that proximal tubule transcripts are reduced while distal tubule transcripts are elevated upon HNF4A loss. Kidney tissues were collected 7 days after tamoxifen injection (n = 4 biologically independent mice). **(b)** Histological staining shows that renal brush border was compromised in $Hnf4\alpha^{KO}$ compared to their littermate controls (n = 4 biologically independent mice, 14 days after tamoxifen injection). **(c)** Kidney weight of $Hnf4\alpha^{KO}$ and their littermate controls. Kidney tissues were weighed 14 days after tamoxifen treatment. The data are presented as mean ± SEM (n = 6 controls and 10 mutants; Student's *t* test, two-sided at $P < 0.01$ **). **(d)** Disorganized brush border was observed in P8 kidney upon HNF4A loss, as evidenced by electron microscopy (Scale bar: 1 um; n = 3 biologically independent mice, tamixifen treatment was done at P2 and P4). The high-magnification images are shown in the boxed insets.

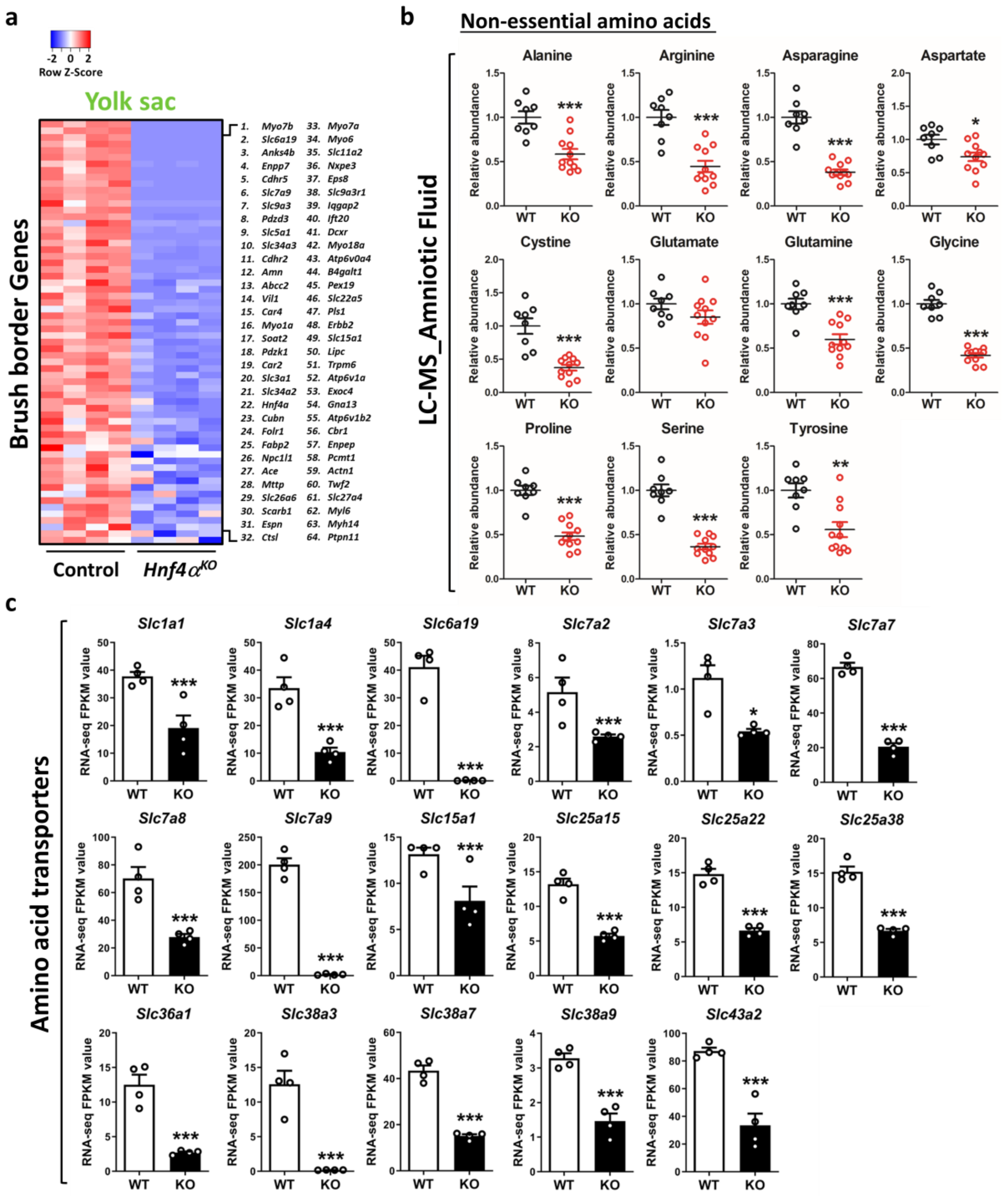

Supplementary Fig 10. Reduced amino acids in the amniotic fluid and compromised amino acid transporter transcripts in the yolk sac are observed upon HNF4A loss. (a) RNA-seq data of brush border genes downregulated upon *Hnf4a* loss (FDR < 0.05, n = 4 biological replicates) are visualized by heatmap. **(b)** In addtion to essential amino acids, non-essential amino acids in the amniotic fluid are also reduced upon *Hnf4a* loss, as evidenced by LC-MS metabolite determination. One mutant sample of amniotic fluid is excluded due to the overall undetectable signals for most of the metabolites (n = 8 WT controls and 11 mutants). The data are presented as mean ± SEM (Student's t test, two-sided at $P < 0.001$ *** and $P < 0.01$ **). **(c)** Amino acid transporter transcripts are compromised upon HNF4A loss, as evidenced by RNA-seq data of E18.5 yolk sac. The data are presented as mean ± SEM (n = 4 WT controls and 4 mutants; Cuffdiff FDR < 0.001***). Schematic of experimental design is shown in **Fig. 5e**.

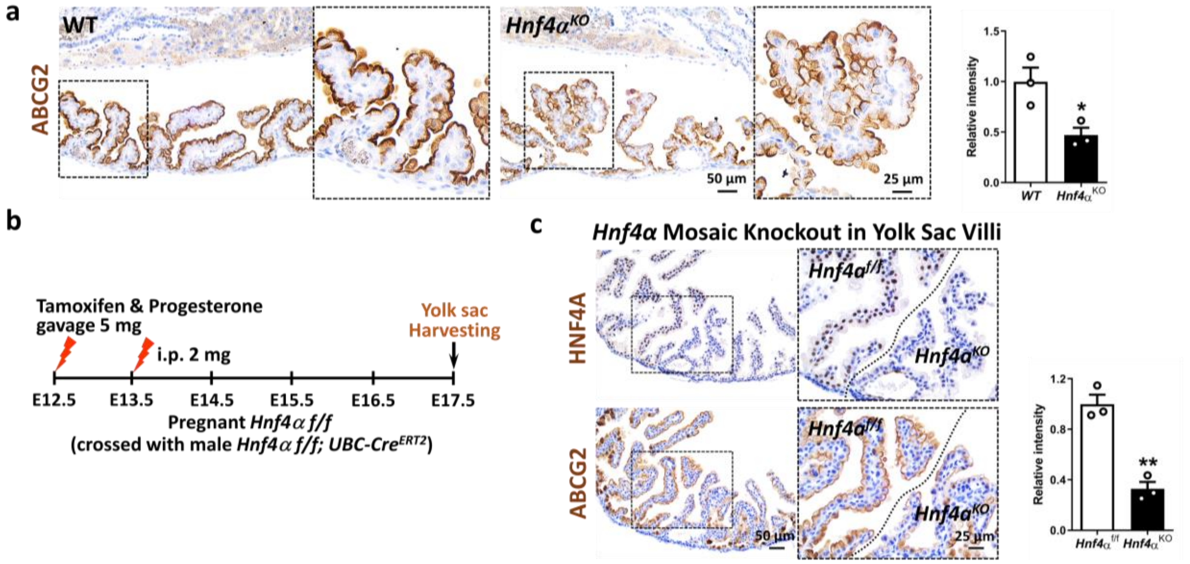

Supplementary Fig 11. IHC staining of ABCG2 in yolk sac. (a) IHC staining and quantification of ABCG2 (brush border marker; uric acid transporter) in the E17.5 yolk sac epithelium (n = 3 biological replicates). Schematic of experimental design is shown in **Fig. 5c**. **(b-c)** IHC staining and quantification of ABCG2 in the mosaic knockout of HNF4A in the E17.5 yolk sac villi (n = 3 biological replicates), see experimental schematic in **(b)**. This experimental design yielded yolk sac tissues with mosaic knockout of HNF4A. Interestingly, the brush border marker ABCG2 positively correlated with expression level of HNF4A in these mosaic tissues, indicating a cell autonomous role for HNF4A in maintaining brush border gene expression in the embryonic yolk sac. In each case, ABCG2 expression is diminished in cells lacking HNF4A, but was preserved in HNF4A-expressing cells. The quantification data are presented as mean ± SEM (n = 3 biological replicates, Student's t test, two-sided at $P < 0.01$ ** and $P < 0.05$ *).

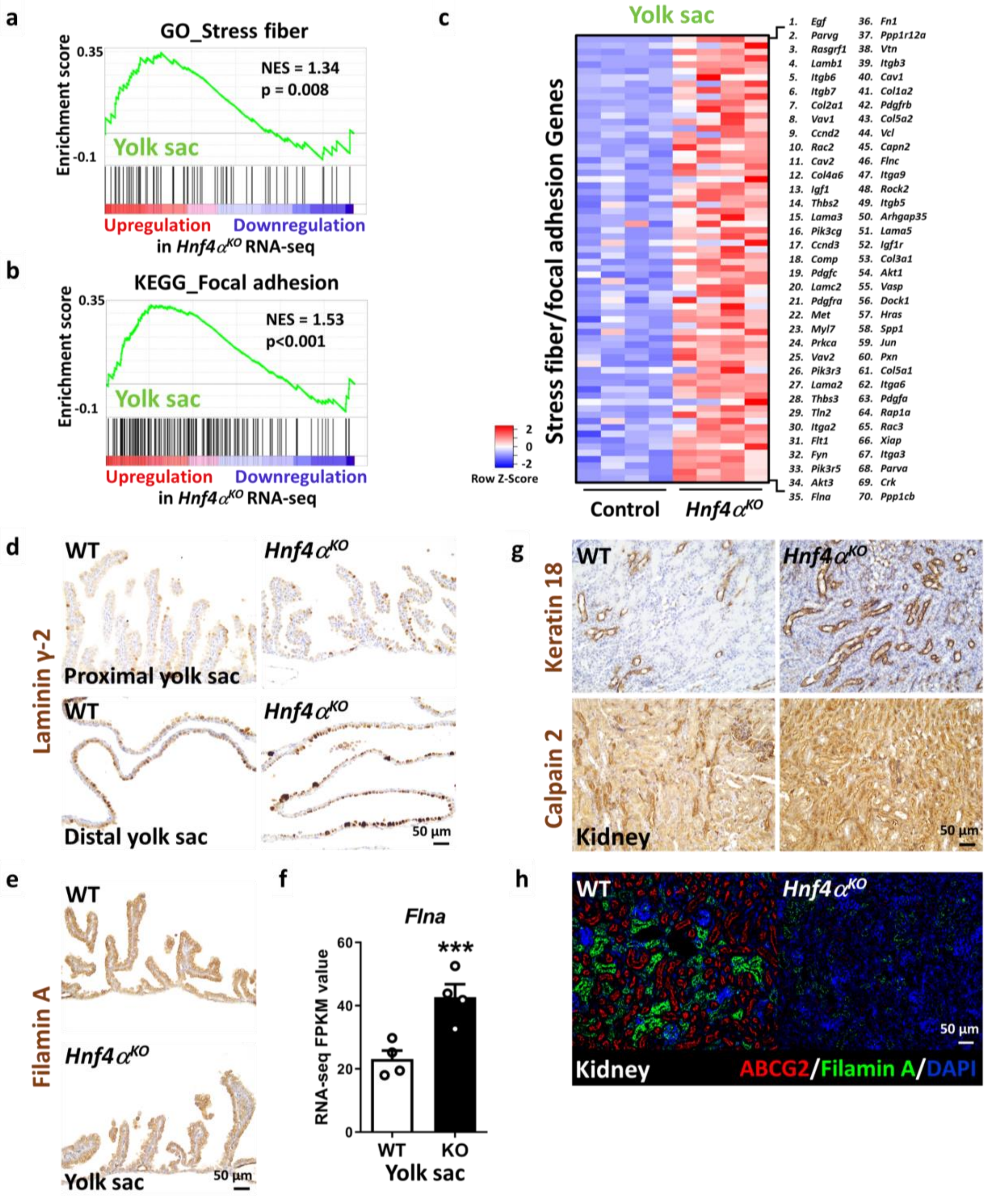

Supplementary Fig 12. The impact of HNF4A loss on stress fiber/focal adhesion in the yolk sac and kidney. (a-f) Yolk sac data of WT vs *Hnf4α^{KO}*. Schematic of experimental design is shown in **Fig. 5e**. GSEA of RNA-seq data from E18.5 yolk sac reveals that **(a)** stress fiber (Kolmogorov-Smirnov test, one-sided for positive and negative enrichment scores, P = 0.008) and **(b)** focal adhesion (Kolmogorov-Smirnov test, one-sided for positive and negative enrichment scores, P < 0.001) gene signatures are all elevated upon HNF4A loss. **(c)** Heatmap of RNA-seq data shows upregulated stress fiber/focal adhesion transcripts upon HNF4A loss in the yolk sac (FDR < 0.05, n = 4 biological replicates). **(d-e)** IHC staining of stress fiber/focal adhesion related proteins (n = 3 biological replicates) in E17.5 yolk sac. Laminin γ-2 (functions in focal adhesion stability) is upregulated upon HNF4A loss in

the distal yolk sac tissues. Transcript levels of Filamin A **(f)** are also upregulated in yolk sac upon HNF4A loss, but not at the protein levels **(e)**. The RNA-seq data are presented as mean ± SEM (n = 3 biological replicates, Cuffdiff FDR < 0.001***). Statistical tests were embedded in Cuffdiff. **(g-h)** Kidney data of WT vs *Hnf4*α^{KO}. **(g)** IHC staining of stress fiber/focal adhesion related proteins (n = 3 biological replicates) in the kidney (n = 3 biological replicates). **(h)** Immunofluorescence co-staining of ABCG2 (brush border marker) and Filamin A (stress fiber marker) in the kidney. ABCG2 marks the proximal tubules in the kidney, whereas Filamin A stains the distal tubules of kidney (n = 3 biological replicates). Unlike intestine and yolk sac, stress fiber related proteins are not detected in the renal brush border-containing cells (proximal tubules).

Supplementary Table 1. SYBR green primer sequences for qRT-PCR

Gene	Forward (5' ---> 3')	Reverse (5' ---> 3')
Hnf4a	GGTCAAGCTACGAGGACAGC	ATGTACTTGGCCCACTCGAC
Hnf4g	GCTGCCAATGATGGTAGTCA	GGTTCTTGTCCAGAGCCTTG
Hprt	TGTTGTTGGATATGCCCTTG	TTGCGCTCATCTTAGGCTTT
