# Supplementary material for: The nuclear receptor HNF4 drives a brush border gene program conserved across murine intestine, kidney, and embryonic yolk sac
Source: Nat Commun. 2021 May 17;12:2886. doi: 10.1038/s41467-021-22761-5 (PMC8129143; doi:10.1038/s41467-021-22761-5)
Supplement: Supplementary file 3 — Description of Additional Supplementary Files [file 41467_2021_22761_MOESM3_ESM.pdf]

Description of Additional Supplementary Files

Title: Supplementary Data 1.
Description: Brush border gene list used for data analysis in this study.

Title: Supplementary Data 2.
Description: HOMER motif analysis of accessible enhancer chromatin regions linked to brush border genes (+/-50 kb). A full table of HOMER analysis related to Fig. 1g.

Title: Supplementary Data 3.
Description: HOMER motif analysis of HNF4 ChIP in intestine and kidney. Full tables of HOMER analysis related to Supplementary Fig. 2b.

Title: Supplementary Data 4.
Description: DiffBind analysis of HNF4A ChIP in Intestine vs Kidney. Full tables of intestine and kidney enriched sites related to Fig. 2a and Supplementary Fig. 2c.

Title: Supplementary Data 5.
Description: Gene ontology analysis of downregulated genes in the intestine and kidney upon HNF4 loss. Full tables of gene ontology analysis related to Fig. 2c.

Title: Supplementary Data 6.
Description: Kidney proximal and distal tubule markers used in this study (derived from kidney scRNA-seq data, Park *et al*., *Science* 2018).
